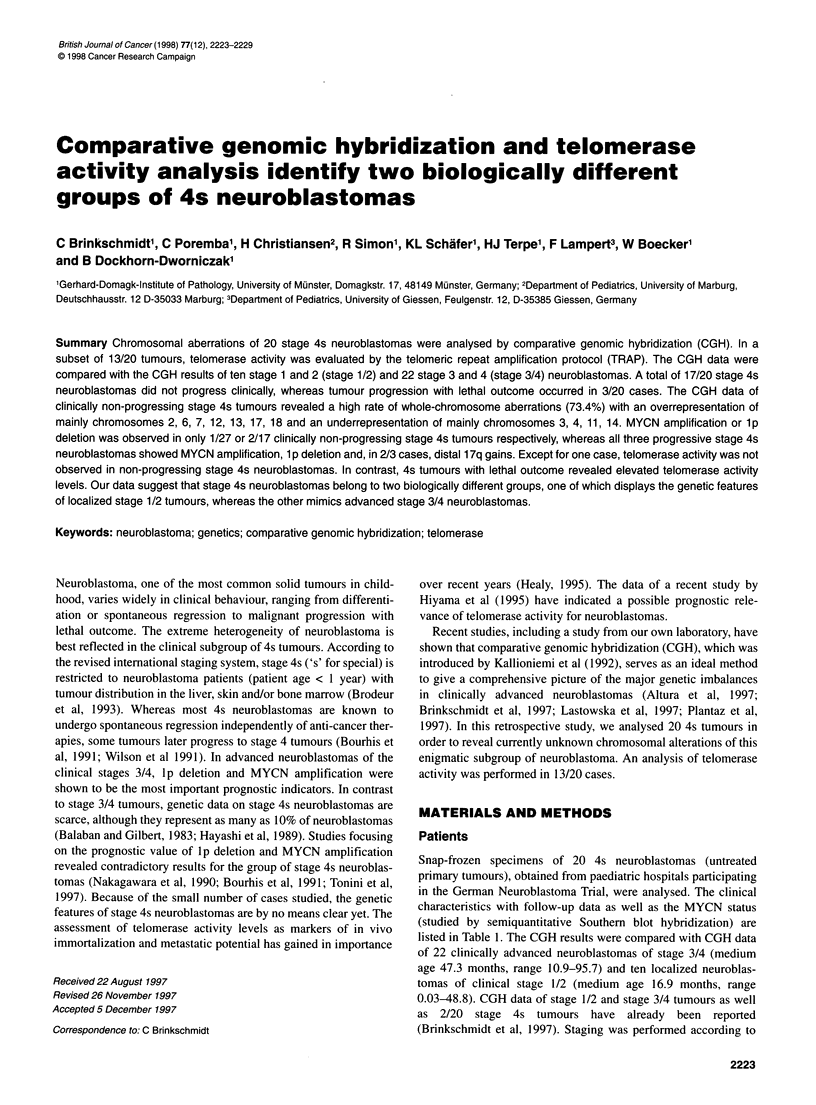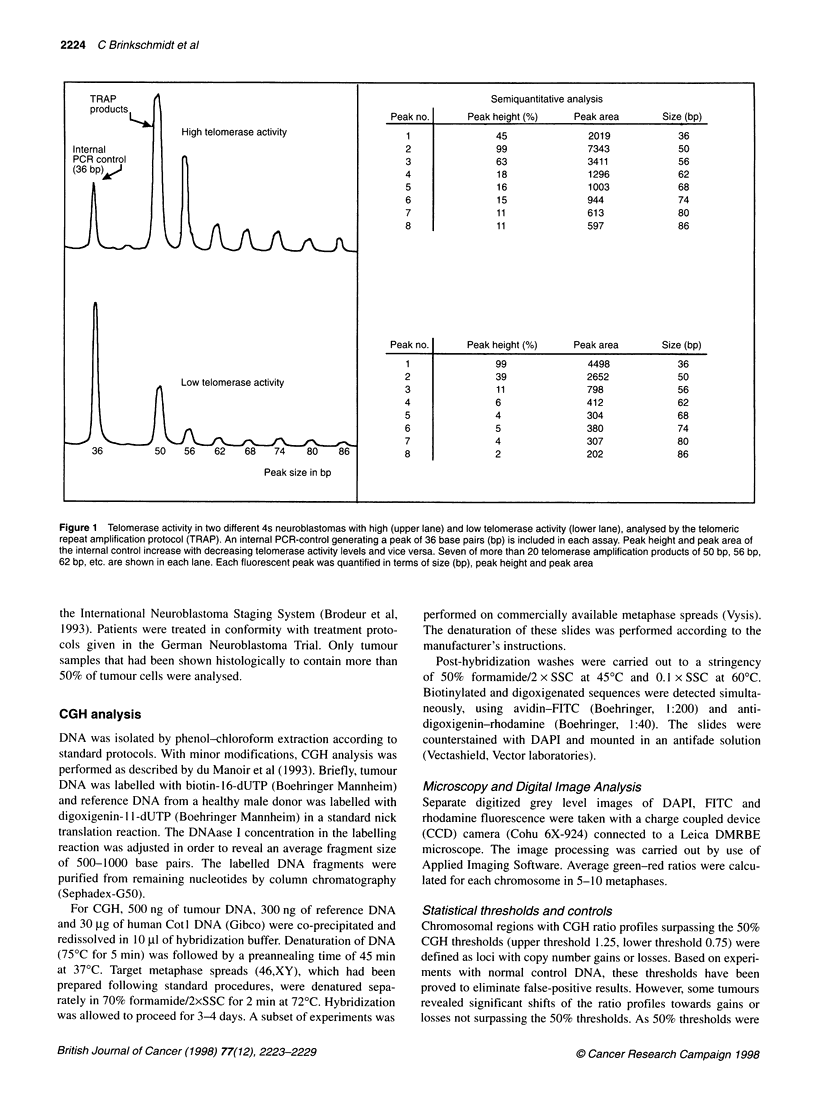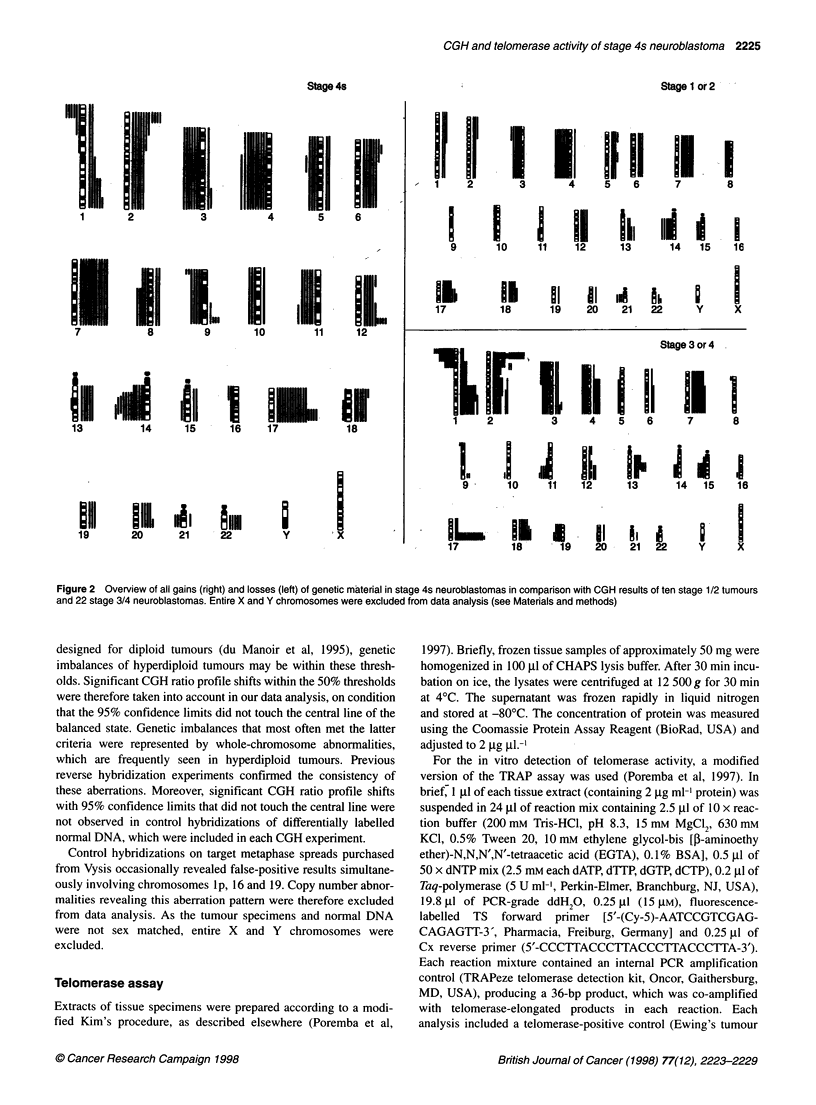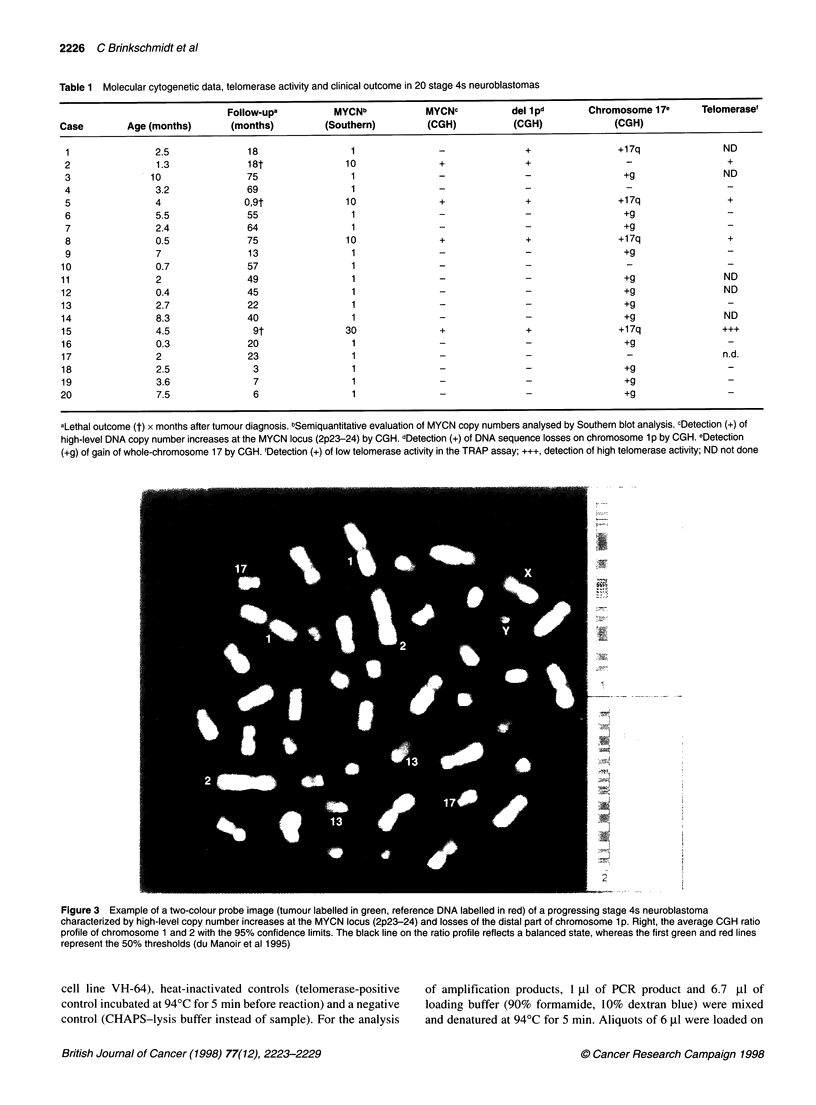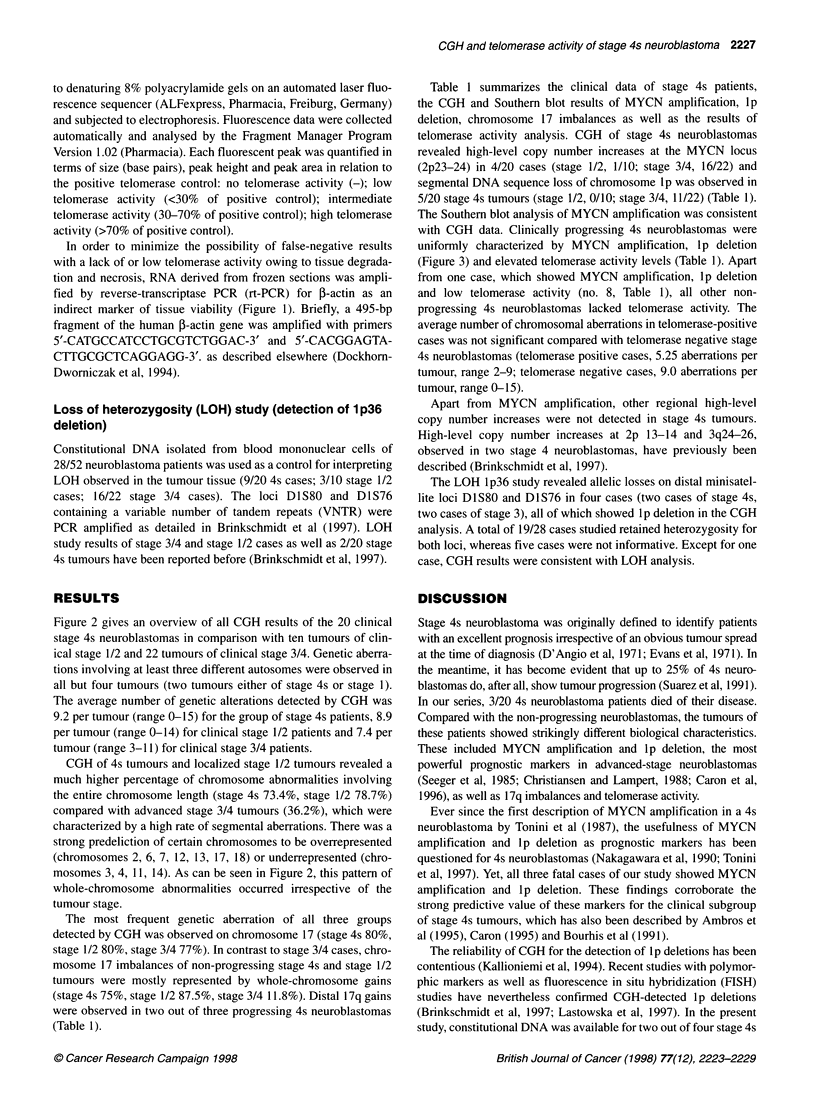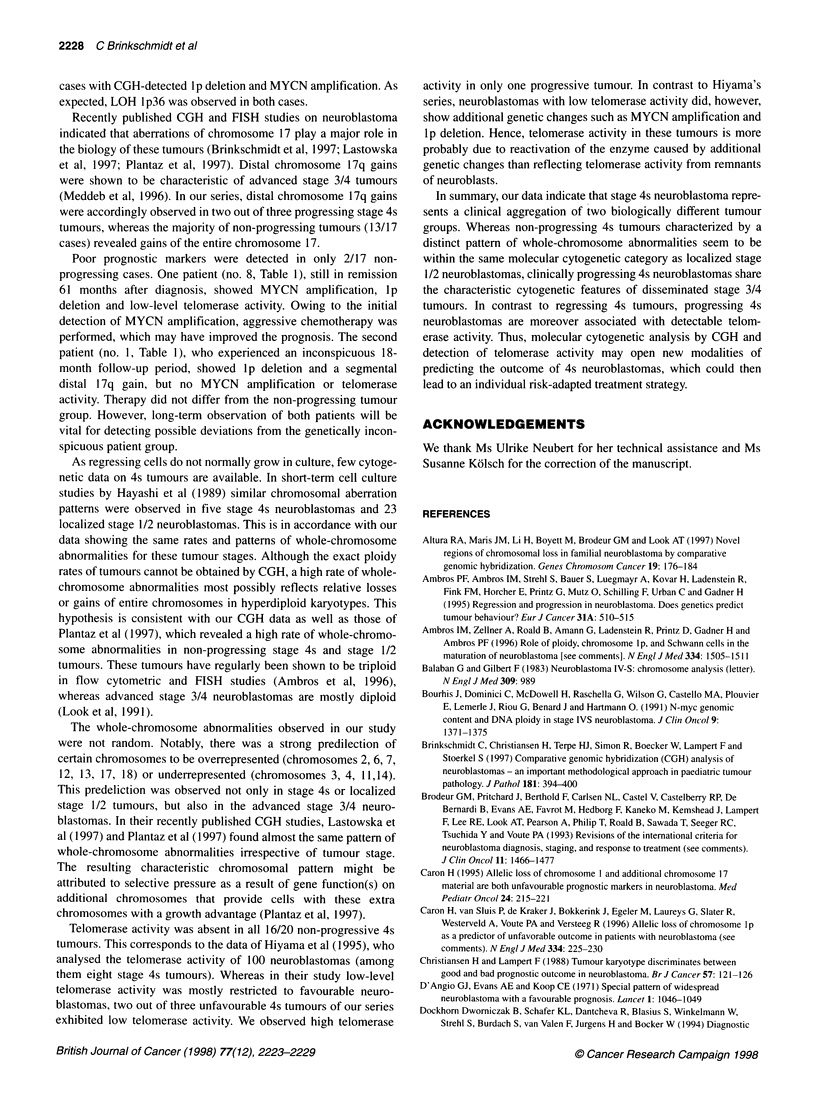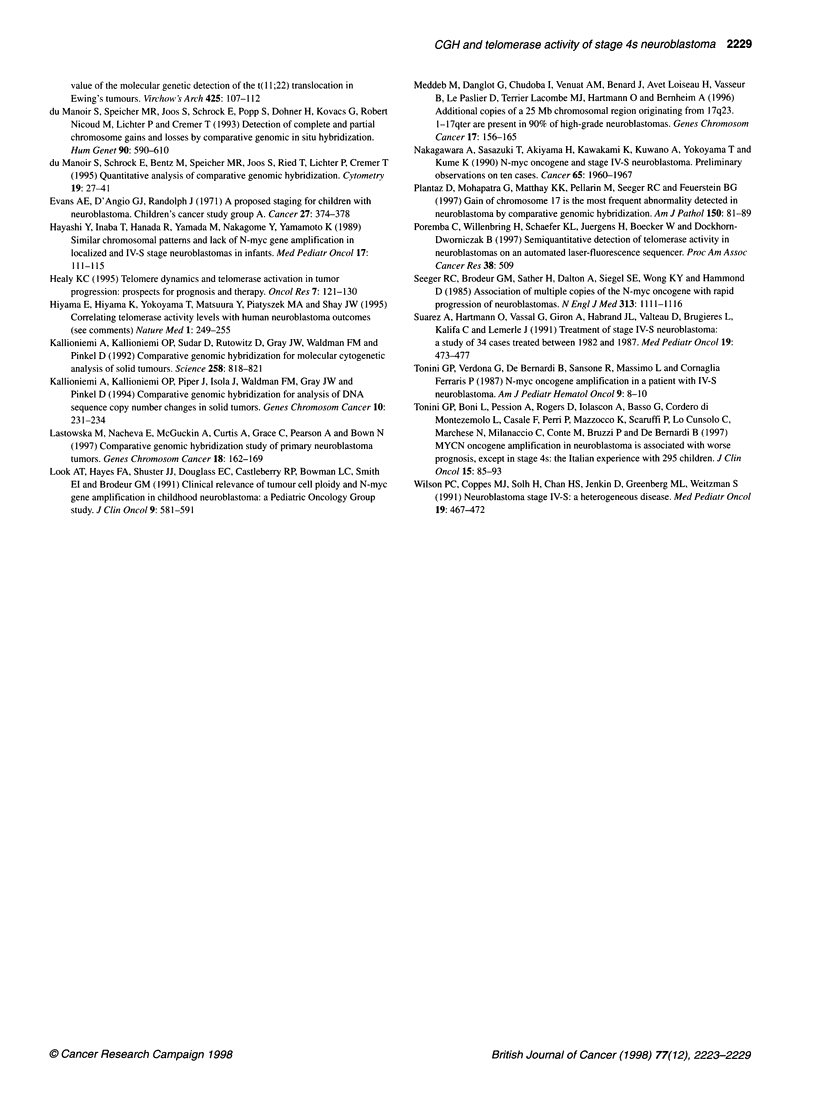# Comparative genomic hybridization and telomerase activity analysis identify two biologically different groups of 4s neuroblastomas.

**DOI:** 10.1038/bjc.1998.370

**Published:** 1998-06

**Authors:** C. Brinkschmidt, C. Poremba, H. Christiansen, R. Simon, K. L. SchÃ¤fer, H. J. Terpe, F. Lampert, W. Boecker, B. Dockhorn-Dworniczak

**Affiliations:** Gerhard-Domagk-Institute of Pathology, University of MÃ¼nster, Germany.

## Abstract

**Images:**


British Journal of Cancer (1998) 77(12), 2223–2229
© 1998 Cancer Research Campaign

### Comparative genomic hybridization and telomerase activity analysis identify two biologically different groups of 4s neuroblastomas

C Brinkschmidt[1], C Poremba[1], H Christiansen[2], R Simon[1], KL Schäfer[1], HJ Terpe[1], F Lampert[3], W Boecker[1] and B Dockhorn-Dworniczak[1]

[1]Gerhard-Domagk-Institute of Pathology, University of Münster, Domagkstr. 17, 48149 Münster, Germany; [2]Department of Pediatrics, University of Marburg, Deutschhausstr. 12 D-35033 Marburg; [3]Department of Pediatrics, University of Giessen, Feulgenstr. 12, D-35385 Giessen, Germany

**Summary** Chromosomal aberrations of 20 stage 4s neuroblastomas were analysed by comparative genomic hybridization (CGH). In a subset of 13/20 tumours, telomerase activity was evaluated by the telomeric repeat amplification protocol (TRAP). The CGH data were compared with the CGH results of ten stage 1 and 2 (stage 1/2) and 22 stage 3 and 4 (stage 3/4) neuroblastomas. A total of 17/20 stage 4s neuroblastomas did not progress clinically, whereas tumour progression with lethal outcome occurred in 3/20 cases. The CGH data of clinically non-progressing stage 4s tumours revealed a high rate of whole-chromosome aberrations (73.4%) with an overrepresentation of mainly chromosomes 2, 6, 7, 12, 13, 17, 18 and an underrepresentation of mainly chromosomes 3, 4, 11, 14. MYCN amplification or 1p deletion was observed in only 1/27 or 2/17 clinically non-progressing stage 4s tumours respectively, whereas all three progressive stage 4s neuroblastomas showed MYCN amplification, 1p deletion and, in 2/3 cases, distal 17q gains. Except for one case, telomerase activity was not observed in non-progressing stage 4s neuroblastomas. In contrast, 4s tumours with lethal outcome revealed elevated telomerase activity levels. Our data suggest that stage 4s neuroblastomas belong to two biologically different groups, one of which displays the genetic features of localized stage 1/2 tumours, whereas the other mimics advanced stage 3/4 neuroblastomas.

**Keywords:** neuroblastoma; genetics; comparative genomic hybridization; telomerase

Neuroblastoma, one of the most common solid tumours in childhood, varies widely in clinical behaviour, ranging from differentiation or spontaneous regression to malignant progression with lethal outcome. The extreme heterogeneity of neuroblastoma is best reflected in the clinical subgroup of 4s tumours. According to the revised international staging system, stage 4s ('s' for special) is restricted to neuroblastoma patients (patient age < 1 year) with tumour distribution in the liver, skin and/or bone marrow (Brodeur et al, 1993). Whereas most 4s neuroblastomas are known to undergo spontaneous regression independently of anti-cancer therapies, some tumours later progress to stage 4 tumours (Bourhis et al, 1991; Wilson et al 1991). In advanced neuroblastomas of the clinical stages 3/4, 1p deletion and MYCN amplification were shown to be the most important prognostic indicators. In contrast to stage 3/4 tumours, genetic data on stage 4s neuroblastomas are scarce, although they represent as many as 10% of neuroblastomas (Balaban and Gilbert, 1983; Hayashi et al, 1989). Studies focusing on the prognostic value of 1p deletion and MYCN amplification revealed contradictory results for the group of stage 4s neuroblastomas (Nakagawara et al, 1990; Bourhis et al, 1991; Tonini et al, 1997). Because of the small number of cases studied, the genetic features of stage 4s neuroblastomas are by no means clear yet. The assessment of telomerase activity levels as markers of in vivo immortalization and metastatic potential has gained in importance over recent years (Healy, 1995). The data of a recent study by Hiyama et al (1995) have indicated a possible prognostic relevance of telomerase activity for neuroblastomas.

Recent studies, including a study from our own laboratory, have shown that comparative genomic hybridization (CGH), which was introduced by Kallioniemi et al (1992), serves as an ideal method to give a comprehensive picture of the major genetic imbalances in clinically advanced neuroblastomas (Altura et al, 1997; Brinkschmidt et al, 1997; Lastowska et al, 1997; Plantaz et al, 1997). In this retrospective study, we analysed 20 4s tumours in order to reveal currently unknown chromosomal alterations of this enigmatic subgroup of neuroblastoma. An analysis of telomerase activity was performed in 13/20 cases.

*Received 22 August 1997*
*Revised 26 November 1997*
*Accepted 5 December 1997*

*Correspondence to:* C Brinkschmidt

### MATERIALS AND METHODS

### Patients

Snap-frozen specimens of 20 4s neuroblastomas (untreated primary tumours), obtained from paediatric hospitals participating in the German Neuroblastoma Trial, were analysed. The clinical characteristics with follow-up data as well as the MYCN status (studied by semiquantitative Southern blot hybridization) are listed in Table 1. The CGH results were compared with CGH data of 22 clinically advanced neuroblastomas of stage 3/4 (medium age 47.3 months, range 10.9–95.7) and ten localized neuroblastomas of clinical stage 1/2 (medium age 16.9 months, range 0.03–48.8). CGH data of stage 1/2 and stage 3/4 tumours as well as 2/20 stage 4s tumours have already been reported (Brinkschmidt et al, 1997). Staging was performed according to

Semiquantitative analysis

| Peak no. | Peak height (%) | Peak area | Size (bp) |
|---|---|---|---|
| 1 | 45 | 2019 | 36 |
| 2 | 99 | 7343 | 50 |
| 3 | 63 | 3411 | 56 |
| 4 | 18 | 1296 | 62 |
| 5 | 16 | 1003 | 68 |
| 6 | 15 | 944 | 74 |
| 7 | 11 | 613 | 80 |
| 8 | 11 | 597 | 86 |

| Peak no. | Peak height (%) | Peak area | Size (bp) |
|---|---|---|---|
| 1 | 99 | 4498 | 36 |
| 2 | 39 | 2652 | 50 |
| 3 | 11 | 798 | 56 |
| 4 | 6 | 412 | 62 |
| 5 | 4 | 304 | 68 |
| 6 | 5 | 380 | 74 |
| 7 | 4 | 307 | 80 |
| 8 | 2 | 202 | 86 |

**Figure 1** Telomerase activity in two different 4s neuroblastomas with high (upper lane) and low telomerase activity (lower lane), analysed by the telomeric repeat amplification protocol (TRAP). An internal PCR-control generating a peak of 36 base pairs (bp) is included in each assay. Peak height and peak area of the internal control increase with decreasing telomerase activity levels and vice versa. Seven of more than 20 telomerase amplification products of 50 bp, 56 bp, 62 bp, etc. are shown in each lane. Each fluorescent peak was quantified in terms of size (bp), peak height and peak area the International Neuroblastoma Staging System (Brodeur et al, 1993). Patients were treated in conformity with treatment protocols given in the German Neuroblastoma Trial. Only tumour samples that had been shown histologically to contain more than 50% of tumour cells were analysed.

### CGH analysis

DNA was isolated by phenol–chloroform extraction according to standard protocols. With minor modifications, CGH analysis was performed as described by du Manoir et al (1993). Briefly, tumour DNA was labelled with biotin-16-dUTP (Boehringer Mannheim) and reference DNA from a healthy male donor was labelled with digoxigenin-11-dUTP (Boehringer Mannheim) in a standard nick translation reaction. The DNAase I concentration in the labelling reaction was adjusted in order to reveal an average fragment size of 500–1000 base pairs. The labelled DNA fragments were purified from remaining nucleotides by column chromatography (Sephadex-G50).

For CGH, 500 ng of tumour DNA, 300 ng of reference DNA and 30 μg of human Cot1 DNA (Gibco) were co-precipitated and redissolved in 10 μl of hybridization buffer. Denaturation of DNA (75°C for 5 min) was followed by a preannealing time of 45 min at 37°C. Target metaphase spreads (46,XY), which had been prepared following standard procedures, were denatured separately in 70% formamide/2×SSC for 2 min at 72°C. Hybridization was allowed to proceed for 3–4 days. A subset of experiments was performed on commercially available metaphase spreads (Vysis). The denaturation of these slides was performed according to the manufacturer's instructions.

Post-hybridization washes were carried out to a stringency of 50% formamide/2 × SSC at 45°C and 0.1 × SSC at 60°C. Biotinylated and digoxigenated sequences were detected simultaneously, using avidin–FITC (Boehringer, 1:200) and anti-digoxigenin–rhodamine (Boehringer, 1:40). The slides were counterstained with DAPI and mounted in an antifade solution (Vectashield, Vector laboratories).

### *Microscopy and Digital Image Analysis*

Separate digitized grey level images of DAPI, FITC and rhodamine fluorescence were taken with a charge coupled device (CCD) camera (Cohu 6X-924) connected to a Leica DMRBE microscope. The image processing was carried out by use of Applied Imaging Software. Average green–red ratios were calculated for each chromosome in 5–10 metaphases.

### *Statistical thresholds and controls*

Chromosomal regions with CGH ratio profiles surpassing the 50% CGH thresholds (upper threshold 1.25, lower threshold 0.75) were defined as loci with copy number gains or losses. Based on experiments with normal control DNA, these thresholds have been proved to eliminate false-positive results. However, some tumours revealed significant shifts of the ratio profiles towards gains or losses not surpassing the 50% thresholds. As 50% thresholds were

© Cancer Research Campaign 1998

**Figure 2** Overview of all gains (right) and losses (left) of genetic material in stage 4s neuroblastomas in comparison with CGH results of ten stage 1/2 tumours and 22 stage 3/4 neuroblastomas. Entire X and Y chromosomes were excluded from data analysis (see Materials and methods)

designed for diploid tumours (du Manoir et al, 1995), genetic imbalances of hyperdiploid tumours may be within these thresholds. Significant CGH ratio profile shifts within the 50% thresholds were therefore taken into account in our data analysis, on condition that the 95% confidence limits did not touch the central line of the balanced state. Genetic imbalances that most often met the latter criteria were represented by whole-chromosome abnormalities, which are frequently seen in hyperdiploid tumours. Previous reverse hybridization experiments confirmed the consistency of these aberrations. Moreover, significant CGH ratio profile shifts with 95% confidence limits that did not touch the central line were not observed in control hybridizations of differentially labelled normal DNA, which were included in each CGH experiment.

Control hybridizations on target metaphase spreads purchased from Vysis occasionally revealed false-positive results simultaneously involving chromosomes 1p, 16 and 19. Copy number abnormalities revealing this aberration pattern were therefore excluded from data analysis. As the tumour specimens and normal DNA were not sex matched, entire X and Y chromosomes were excluded.

### Telomerase assay

Extracts of tissue specimens were prepared according to a modified Kim's procedure, as described elsewhere (Poremba et al, 1997). Briefly, frozen tissue samples of approximately 50 mg were homogenized in 100 µl of CHAPS lysis buffer. After 30 min incubation on ice, the lysates were centrifuged at 12 500 ***g*** for 30 min at 4°C. The supernatant was frozen rapidly in liquid nitrogen and stored at –80°C. The concentration of protein was measured using the Coomassie Protein Assay Reagent (BioRad, USA) and adjusted to 2 µg $\mu l^{-1}$.

For the in vitro detection of telomerase activity, a modified version of the TRAP assay was used (Poremba et al, 1997). In brief, 1 µl of each tissue extract (containing 2 µg $ml^{-1}$ protein) was suspended in 24 µl of reaction mix containing 2.5 µl of 10 × reaction buffer (200 mM Tris-HCl, pH 8.3, 15 mM $MgCl_2$, 630 mM KCl, 0.5% Tween 20, 10 mM ethylene glycol-bis [β-aminoethy ether)-N,N,N′,N′-tetraacetic acid (EGTA), 0.1% BSA], 0.5 µl of 50 × dNTP mix (2.5 mM each dATP, dTTP, dGTP, dCTP), 0.2 µl of *Taq*-polymerase (5 U $ml^{-1}$, Perkin-Elmer, Branchburg, NJ, USA), 19.8 µl of PCR-grade $ddH_2O$, 0.25 µl (15 µM), fluorescence-labelled TS forward primer [5′-(Cy-5)-AATCCGTCGAG-CAGAGTT-3′, Pharmacia, Freiburg, Germany] and 0.25 µl of Cx reverse primer (5′-CCCTTACCCTTACCCTTACCCTTA-3′). Each reaction mixture contained an internal PCR amplification control (TRAPeze telomerase detection kit, Oncor, Gaithersburg, MD, USA), producing a 36-bp product, which was co-amplified with telomerase-elongated products in each reaction. Each analysis included a telomerase-positive control (Ewing's tumour

© Cancer Research Campaign 1998

**Table 1** Molecular cytogenetic data, telomerase activity and clinical outcome in 20 stage 4s neuroblastomas

| Case | Age (months) | Follow-up[a] (months) | MYCN[b] (Southern) | MYCN[c] (CGH) | del 1p[d] (CGH) | Chromosome 17[e] (CGH) | Telomerase[f] |
|---|---|---|---|---|---|---|---|
| 1 | 2.5 | 18 | 1 | – | + | +17q | ND |
| 2 | 1.3 | 18† | 10 | + | + | – | + |
| 3 | 10 | 75 | 1 | – | – | +g | ND |
| 4 | 3.2 | 69 | 1 | – | – | – | – |
| 5 | 4 | 0,9† | 10 | + | + | +17q | + |
| 6 | 5.5 | 55 | 1 | – | – | +g | – |
| 7 | 2.4 | 64 | 1 | – | – | +g | – |
| 8 | 0.5 | 75 | 10 | + | + | +17q | + |
| 9 | 7 | 13 | 1 | – | – | +g | – |
| 10 | 0.7 | 57 | 1 | – | – | – | – |
| 11 | 2 | 49 | 1 | – | – | +g | ND |
| 12 | 0.4 | 45 | 1 | – | – | +g | ND |
| 13 | 2.7 | 22 | 1 | – | – | +g | – |
| 14 | 8.3 | 40 | 1 | – | – | +g | ND |
| 15 | 4.5 | 9† | 30 | + | + | +17q | +++ |
| 16 | 0.3 | 20 | 1 | – | – | +g | – |
| 17 | 2 | 23 | 1 | – | – | – | n.d. |
| 18 | 2.5 | 3 | 1 | – | – | +g | – |
| 19 | 3.6 | 7 | 1 | – | – | +g | – |
| 20 | 7.5 | 6 | 1 | – | – | +g | – |

[a]Lethal outcome (†) × months after tumour diagnosis. [b]Semiquantitative evaluation of MYCN copy numbers analysed by Southern blot analysis. [c]Detection (+) of high-level DNA copy number increases at the MYCN locus (2p23–24) by CGH. [d]Detection (+) of DNA sequence losses on chromosome 1p by CGH. [e]Detection (+g) of gain of whole-chromosome 17 by CGH. [f]Detection (+) of low telomerase activity in the TRAP assay; +++, detection of high telomerase activity; ND not done

**Figure 3** Example of a two-colour probe image (tumour labelled in green, reference DNA labelled in red) of a progressing stage 4s neuroblastoma characterized by high-level copy number increases at the MYCN locus (2p23–24) and losses of the distal part of chromosome 1p. Right, the average CGH ratio profile of chromosome 1 and 2 with the 95% confidence limits. The black line on the ratio profile reflects a balanced state, whereas the first green and red lines represent the 50% thresholds (du Manoir et al 1995)

cell line VH-64), heat-inactivated controls (telomerase-positive control incubated at 94°C for 5 min before reaction) and a negative control (CHAPS–lysis buffer instead of sample). For the analysis of amplification products, 1 μl of PCR product and 6.7 μl of loading buffer (90% formamide, 10% dextran blue) were mixed and denatured at 94°C for 5 min. Aliquots of 6 μl were loaded on

*© Cancer Research Campaign 1998*

to denaturing 8% polyacrylamide gels on an automated laser fluorescence sequencer (ALFexpress, Pharmacia, Freiburg, Germany) and subjected to electrophoresis. Fluorescence data were collected automatically and analysed by the Fragment Manager Program Version 1.02 (Pharmacia). Each fluorescent peak was quantified in terms of size (base pairs), peak height and peak area in relation to the positive telomerase control: no telomerase activity (–); low telomerase activity (<30% of positive control); intermediate telomerase activity (30–70% of positive control); high telomerase activity (>70% of positive control).

In order to minimize the possibility of false-negative results with a lack of or low telomerase activity owing to tissue degradation and necrosis, RNA derived from frozen sections was amplified by reverse-transcriptase PCR (rt-PCR) for β-actin as an indirect marker of tissue viability (Figure 1). Briefly, a 495-bp fragment of the human β-actin gene was amplified with primers 5′-CATGCCATCCTGCGTCTGGAC-3′ and 5′-CACGGAGTA-CTTGCGCTCAGGAGG-3′. as described elsewhere (Dockhorn-Dworniczak et al, 1994).

### Loss of heterozygosity (LOH) study (detection of 1p36 deletion)

Constitutional DNA isolated from blood mononuclear cells of 28/52 neuroblastoma patients was used as a control for interpreting LOH observed in the tumour tissue (9/20 4s cases; 3/10 stage 1/2 cases; 16/22 stage 3/4 cases). The loci D1S80 and D1S76 containing a variable number of tandem repeats (VNTR) were PCR amplified as detailed in Brinkschmidt et al (1997). LOH study results of stage 3/4 and stage 1/2 cases as well as 2/20 stage 4s tumours have been reported before (Brinkschmidt et al, 1997).

### RESULTS

Figure 2 gives an overview of all CGH results of the 20 clinical stage 4s neuroblastomas in comparison with ten tumours of clinical stage 1/2 and 22 tumours of clinical stage 3/4. Genetic aberrations involving at least three different autosomes were observed in all but four tumours (two tumours either of stage 4s or stage 1). The average number of genetic alterations detected by CGH was 9.2 per tumour (range 0–15) for the group of stage 4s patients, 8.9 per tumour (range 0–14) for clinical stage 1/2 patients and 7.4 per tumour (range 3–11) for clinical stage 3/4 patients.

CGH of 4s tumours and localized stage 1/2 tumours revealed a much higher percentage of chromosome abnormalities involving the entire chromosome length (stage 4s 73.4%, stage 1/2 78.7%) compared with advanced stage 3/4 tumours (36.2%), which were characterized by a high rate of segmental aberrations. There was a strong predeliction of certain chromosomes to be overrepresented (chromosomes 2, 6, 7, 12, 13, 17, 18) or underrepresented (chromosomes 3, 4, 11, 14). As can be seen in Figure 2, this pattern of whole-chromosome abnormalities occurred irrespective of the tumour stage.

The most frequent genetic aberration of all three groups detected by CGH was observed on chromosome 17 (stage 4s 80%, stage 1/2 80%, stage 3/4 77%). In contrast to stage 3/4 cases, chromosome 17 imbalances of non-progressing stage 4s and stage 1/2 tumours were mostly represented by whole-chromosome gains (stage 4s 75%, stage 1/2 87.5%, stage 3/4 11.8%). Distal 17q gains were observed in two out of three progressing 4s neuroblastomas (Table 1).

Table 1 summarizes the clinical data of stage 4s patients, the CGH and Southern blot results of MYCN amplification, 1p deletion, chromosome 17 imbalances as well as the results of telomerase activity analysis. CGH of stage 4s neuroblastomas revealed high-level copy number increases at the MYCN locus (2p23–24) in 4/20 cases (stage 1/2, 1/10; stage 3/4, 16/22) and segmental DNA sequence loss of chromosome 1p was observed in 5/20 stage 4s tumours (stage 1/2, 0/10; stage 3/4, 11/22) (Table 1). The Southern blot analysis of MYCN amplification was consistent with CGH data. Clinically progressing 4s neuroblastomas were uniformly characterized by MYCN amplification, 1p deletion (Figure 3) and elevated telomerase activity levels (Table 1). Apart from one case, which showed MYCN amplification, 1p deletion and low telomerase activity (no. 8, Table 1), all other non-progressing 4s neuroblastomas lacked telomerase activity. The average number of chromosomal aberrations in telomerase-positive cases was not significant compared with telomerase negative stage 4s neuroblastomas (telomerase positive cases, 5.25 aberrations per tumour, range 2–9; telomerase negative cases, 9.0 aberrations per tumour, range 0–15).

Apart from MYCN amplification, other regional high-level copy number increases were not detected in stage 4s tumours. High-level copy number increases at 2p 13–14 and 3q24–26, observed in two stage 4 neuroblastomas, have previously been described (Brinkschmidt et al, 1997).

The LOH 1p36 study revealed allelic losses on distal minisatellite loci D1S80 and D1S76 in four cases (two cases of stage 4s, two cases of stage 3), all of which showed 1p deletion in the CGH analysis. A total of 19/28 cases studied retained heterozygosity for both loci, whereas five cases were not informative. Except for one case, CGH results were consistent with LOH analysis.

### DISCUSSION

Stage 4s neuroblastoma was originally defined to identify patients with an excellent prognosis irrespective of an obvious tumour spread at the time of diagnosis (D'Angio et al, 1971; Evans et al, 1971). In the meantime, it has become evident that up to 25% of 4s neuroblastomas do, after all, show tumour progression (Suarez et al, 1991). In our series, 3/20 4s neuroblastoma patients died of their disease. Compared with the non-progressing neuroblastomas, the tumours of these patients showed strikingly different biological characteristics. These included MYCN amplification and 1p deletion, the most powerful prognostic markers in advanced-stage neuroblastomas (Seeger et al, 1985; Christiansen and Lampert, 1988; Caron et al, 1996), as well as 17q imbalances and telomerase activity.

Ever since the first description of MYCN amplification in a 4s neuroblastoma by Tonini et al (1987), the usefulness of MYCN amplification and 1p deletion as prognostic markers has been questioned for 4s neuroblastomas (Nakagawara et al, 1990; Tonini et al, 1997). Yet, all three fatal cases of our study showed MYCN amplification and 1p deletion. These findings corroborate the strong predictive value of these markers for the clinical subgroup of stage 4s tumours, which has also been described by Ambros et al (1995), Caron (1995) and Bourhis et al (1991).

The reliability of CGH for the detection of 1p deletions has been contentious (Kallioniemi et al, 1994). Recent studies with polymorphic markers as well as fluorescence in situ hybridization (FISH) studies have nevertheless confirmed CGH-detected 1p deletions (Brinkschmidt et al, 1997; Lastowska et al, 1997). In the present study, constitutional DNA was available for two out of four stage 4s

© Cancer Research Campaign 1998

cases with CGH-detected 1p deletion and MYCN amplification. As expected, LOH 1p36 was observed in both cases.

Recently published CGH and FISH studies on neuroblastoma indicated that aberrations of chromosome 17 play a major role in the biology of these tumours (Brinkschmidt et al, 1997; Lastowska et al, 1997; Plantaz et al, 1997). Distal chromosome 17q gains were shown to be characteristic of advanced stage 3/4 tumours (Meddeb et al, 1996). In our series, distal chromosome 17q gains were accordingly observed in two out of three progressing stage 4s tumours, whereas the majority of non-progressing tumours (13/17 cases) revealed gains of the entire chromosome 17.

Poor prognostic markers were detected in only 2/17 non-progressing cases. One patient (no. 8, Table 1), still in remission 61 months after diagnosis, showed MYCN amplification, 1p deletion and low-level telomerase activity. Owing to the initial detection of MYCN amplification, aggressive chemotherapy was performed, which may have improved the prognosis. The second patient (no. 1, Table 1), who experienced an inconspicuous 18-month follow-up period, showed 1p deletion and a segmental distal 17q gain, but no MYCN amplification or telomerase activity. Therapy did not differ from the non-progressing tumour group. However, long-term observation of both patients will be vital for detecting possible deviations from the genetically inconspicuous patient group.

As regressing cells do not normally grow in culture, few cytogenetic data on 4s tumours are available. In short-term cell culture studies by Hayashi et al (1989) similar chromosomal aberration patterns were observed in five stage 4s neuroblastomas and 23 localized stage 1/2 neuroblastomas. This is in accordance with our data showing the same rates and patterns of whole-chromosome abnormalities for these tumour stages. Although the exact ploidy rates of tumours cannot be obtained by CGH, a high rate of whole-chromosome abnormalities most possibly reflects relative losses or gains of entire chromosomes in hyperdiploid karyotypes. This hypothesis is consistent with our CGH data as well as those of Plantaz et al (1997), which revealed a high rate of whole-chromosome abnormalities in non-progressing stage 4s and stage 1/2 tumours. These tumours have regularly been shown to be triploid in flow cytometric and FISH studies (Ambros et al, 1996), whereas advanced stage 3/4 neuroblastomas are mostly diploid (Look et al, 1991).

The whole-chromosome abnormalities observed in our study were not random. Notably, there was a strong predilection of certain chromosomes to be overrepresented (chromosomes 2, 6, 7, 12, 13, 17, 18) or underrepresented (chromosomes 3, 4, 11,14). This predeliction was observed not only in stage 4s or localized stage 1/2 tumours, but also in the advanced stage 3/4 neuroblastomas. In their recently published CGH studies, Lastowska et al (1997) and Plantaz et al (1997) found almost the same pattern of whole-chromosome abnormalities irrespective of tumour stage. The resulting characteristic chromosomal pattern might be attributed to selective pressure as a result of gene function(s) on additional chromosomes that provide cells with these extra chromosomes with a growth advantage (Plantaz et al, 1997).

Telomerase activity was absent in all 16/20 non-progressive 4s tumours. This corresponds to the data of Hiyama et al (1995), who analysed the telomerase activity of 100 neuroblastomas (among them eight stage 4s tumours). Whereas in their study low-level telomerase activity was mostly restricted to favourable neuroblastomas, two out of three unfavourable 4s tumours of our series exhibited low telomerase activity. We observed high telomerase activity in only one progressive tumour. In contrast to Hiyama's series, neuroblastomas with low telomerase activity did, however, show additional genetic changes such as MYCN amplification and 1p deletion. Hence, telomerase activity in these tumours is more probably due to reactivation of the enzyme caused by additional genetic changes than reflecting telomerase activity from remnants of neuroblasts.

In summary, our data indicate that stage 4s neuroblastoma represents a clinical aggregation of two biologically different tumour groups. Whereas non-progressing 4s tumours characterized by a distinct pattern of whole-chromosome abnormalities seem to be within the same molecular cytogenetic category as localized stage 1/2 neuroblastomas, clinically progressing 4s neuroblastomas share the characteristic cytogenetic features of disseminated stage 3/4 tumours. In contrast to regressing 4s tumours, progressing 4s neuroblastomas are moreover associated with detectable telomerase activity. Thus, molecular cytogenetic analysis by CGH and detection of telomerase activity may open new modalities of predicting the outcome of 4s neuroblastomas, which could then lead to an individual risk-adapted treatment strategy.

### ACKNOWLEDGEMENTS

We thank Ms Ulrike Neubert for her technical assistance and Ms Susanne Kölsch for the correction of the manuscript.

### REFERENCES

Altura RA, Maris JM, Li H, Boyett M, Brodeur GM and Look AT (1997) Novel regions of chromosomal loss in familial neuroblastoma by comparative genomic hybridization. *Genes Chromosom Cancer* **19**: 176–184

Ambros PF, Ambros IM, Strehl S, Bauer S, Luegmayr A, Kovar H, Ladenstein R, Fink FM, Horcher E, Printz G, Mutz O, Schilling F, Urban C and Gadner H (1995) Regression and progression in neuroblastoma. Does genetics predict tumour behaviour? *Eur J Cancer* **31A**: 510–515

Ambros IM, Zellner A, Roald B, Amann G, Ladenstein R, Printz D, Gadner H and Ambros PF (1996) Role of ploidy, chromosome 1p, and Schwann cells in the maturation of neuroblastoma [see comments]. *N Engl J Med* **334**: 1505–1511

Balaban G and Gilbert F (1983) Neuroblastoma IV-S: chromosome analysis (letter). *N Engl J Med* **309**: 989

Bourhis J, Dominici C, McDowell H, Raschella G, Wilson G, Castello MA, Plouvier E, Lemerle J, Riou G, Benard J and Hartmann O. (1991) N-myc genomic content and DNA ploidy in stage IVS neuroblastoma. *J Clin Oncol* **9**: 1371–1375

Brinkschmidt C, Christiansen H, Terpe HJ, Simon R, Boecker W, Lampert F and Stoerkel S (1997) Comparative genomic hybridization (CGH) analysis of neuroblastomas – an important methodological approach in paediatric tumour pathology. *J Pathol* **181**: 394–400

Brodeur GM, Pritchard J, Berthold F, Carlsen NL, Castel V, Castelberry RP, De Bernardi B, Evans AE, Favrot M, Hedborg F, Kaneko M, Kemshead J, Lampert F, Lee RE, Look AT, Pearson A, Philip T, Roald B, Sawada T, Seeger RC, Tsuchida Y and Voute PA (1993) Revisions of the international criteria for neuroblastoma diagnosis, staging, and response to treatment (see comments). *J Clin Oncol* **11**: 1466–1477

Caron H (1995) Allelic loss of chromosome 1 and additional chromosome 17 material are both unfavourable prognostic markers in neuroblastoma. *Med Pediatr Oncol* **24**: 215–221

Caron H, van Sluis P, de Kraker J, Bokkerink J, Egeler M, Laureys G, Slater R, Westerveld A, Voute PA and Versteeg R (1996) Allelic loss of chromosome 1p as a predictor of unfavorable outcome in patients with neuroblastoma (see comments). *N Engl J Med* **334**: 225–230

Christiansen H and Lampert F (1988) Tumour karyotype discriminates between good and bad prognostic outcome in neuroblastoma. *Br J Cancer* **57**: 121–126

D'Angio GJ, Evans AE and Koop CE (1971) Special pattern of widespread neuroblastoma with a favourable prognosis. *Lancet* **1**: 1046–1049

Dockhorn Dworniczak B, Schafer KL, Dantcheva R, Blasius S, Winkelmann W, Strehl S, Burdach S, van Valen F, Jurgens H and Bocker W (1994) Diagnostic

 © Cancer Research Campaign 1998

value of the molecular genetic detection of the t(11;22) translocation in Ewing's tumours. *Virchow's Arch* **425**: 107–112

du Manoir S, Speicher MR, Joos S, Schrock E, Popp S, Dohner H, Kovacs G, Robert Nicoud M, Lichter P and Cremer T (1993) Detection of complete and partial chromosome gains and losses by comparative genomic in situ hybridization. *Hum Genet* **90**: 590–610

du Manoir S, Schrock E, Bentz M, Speicher MR, Joos S, Ried T, Lichter P, Cremer T (1995) Quantitative analysis of comparative genomic hybridization. *Cytometry* **19**: 27–41

Evans AE, D'Angio GJ, Randolph J (1971) A proposed staging for children with neuroblastoma. Children's cancer study group A. *Cancer* **27**: 374–378

Hayashi Y, Inaba T, Hanada R, Yamada M, Nakagome Y, Yamamoto K (1989) Similar chromosomal patterns and lack of N-myc gene amplification in localized and IV-S stage neuroblastomas in infants. *Med Pediatr Oncol* **17**: 111–115

Healy KC (1995) Telomere dynamics and telomerase activation in tumor progression: prospects for prognosis and therapy. *Oncol Res* **7**: 121–130

Hiyama E, Hiyama K, Yokoyama T, Matsuura Y, Piatyszek MA and Shay JW (1995) Correlating telomerase activity levels with human neuroblastoma outcomes (see comments) *Nature Med* **1**: 249–255

Kallioniemi A, Kallioniemi OP, Sudar D, Rutowitz D, Gray JW, Waldman FM and Pinkel D (1992) Comparative genomic hybridization for molecular cytogenetic analysis of solid tumours. *Science* **258**: 818–821

Kallioniemi A, Kallioniemi OP, Piper J, Isola J, Waldman FM, Gray JW and Pinkel D (1994) Comparative genomic hybridization for analysis of DNA sequence copy number changes in solid tumors. *Genes Chromosom Cancer* **10**: 231–234

Lastowska M, Nacheva E, McGuckin A, Curtis A, Grace C, Pearson A and Bown N (1997) Comparative genomic hybridization study of primary neuroblastoma tumors. *Genes Chromosom Cancer* **18**: 162–169

Look AT, Hayes FA, Shuster JJ, Douglass EC, Castleberry RP, Bowman LC, Smith EI and Brodeur GM (1991) Clinical relevance of tumour cell ploidy and N-myc gene amplification in childhood neuroblastoma: a Pediatric Oncology Group study. *J Clin Oncol* **9**: 581–591

Meddeb M, Danglot G, Chudoba I, Venuat AM, Benard J, Avet Loiseau H, Vasseur B, Le Paslier D, Terrier Lacombe MJ, Hartmann O and Bernheim A (1996) Additional copies of a 25 Mb chromosomal region originating from 17q23. 1–17qter are present in 90% of high-grade neuroblastomas. *Genes Chromosom Cancer* **17**: 156–165

Nakagawara A, Sasazuki T, Akiyama H, Kawakami K, Kuwano A, Yokoyama T and Kume K (1990) N-myc oncogene and stage IV-S neuroblastoma. Preliminary observations on ten cases. *Cancer* **65**: 1960–1967

Plantaz D, Mohapatra G, Matthay KK, Pellarin M, Seeger RC and Feuerstein BG (1997) Gain of chromosome 17 is the most frequent abnormality detected in neuroblastoma by comparative genomic hybridization. *Am J Pathol* **150**: 81–89

Poremba C, Willenbring H, Schaefer KL, Juergens H, Boecker W and Dockhorn-Dworniczak B (1997) Semiquantitative detection of telomerase activity in neuroblastomas on an automated laser-fluorescence sequencer. *Proc Am Assoc Cancer Res* **38**: 509

Seeger RC, Brodeur GM, Sather H, Dalton A, Siegel SE, Wong KY and Hammond D (1985) Association of multiple copies of the N-myc oncogene with rapid progression of neuroblastomas. *N Engl J Med* **313**: 1111–1116

Suarez A, Hartmann O, Vassal G, Giron A, Habrand JL, Valteau D, Brugieres L, Kalifa C and Lemerle J (1991) Treatment of stage IV-S neuroblastoma: a study of 34 cases treated between 1982 and 1987. *Med Pediatr Oncol* **19**: 473–477

Tonini GP, Verdona G, De Bernardi B, Sansone R, Massimo L and Cornaglia Ferraris P (1987) N-myc oncogene amplification in a patient with IV-S neuroblastoma. *Am J Pediatr Hematol Oncol* **9**: 8–10

Tonini GP, Boni L, Pession A, Rogers D, Iolascon A, Basso G, Cordero di Montezemolo L, Casale F, Perri P, Mazzocco K, Scaruffi P, Lo Cunsolo C, Marchese N, Milanaccio C, Conte M, Bruzzi P and De Bernardi B (1997) MYCN oncogene amplification in neuroblastoma is associated with worse prognosis, except in stage 4s: the Italian experience with 295 children. *J Clin Oncol* **15**: 85–93

Wilson PC, Coppes MJ, Solh H, Chan HS, Jenkin D, Greenberg ML, Weitzman S (1991) Neuroblastoma stage IV-S: a heterogeneous disease. *Med Pediatr Oncol* **19**: 467–472

© Cancer Research Campaign 1998